# Children born preterm admitted to paediatric intensive care for bronchiolitis: a systematic review and meta-analysis

**DOI:** 10.1186/s12887-023-04150-7

**Published:** 2023-06-29

**Authors:** Tim J. van Hasselt, Kirstin Webster, Chris Gale, Elizabeth S. Draper, Sarah E. Seaton

**Affiliations:** 1 Department of Population Health Sciences, University of Leicester, University Rd, Leicester, LE1 7RH UK; 2 Neonatal Medicine, School of Public Health, Faculty of Medicine, Chelsea and Westminster Hospital Campus, Imperial College London, London, UK

**Keywords:** Intensive care units, Pediatric, Premature birth, Infant, Premature, Bronchiolitis, Mechanical ventilation, Hospital mortality

## Abstract

**Background:**

To undertake a systematic review of studies describing the proportion of children admitted to a paediatric intensive care unit (PICU) for respiratory syncytial virus (RSV) and/or bronchiolitis who were born preterm, and compare their outcomes in PICU with children born at term.

**Methods:**

We searched Medline, Embase and Scopus. Citations and references of included articles were searched**.** We included studies published from the year 2000 onwards, from high-income countries, that examined children 0–18 years of age, admitted to PICU from the year 2000 onwards for RSV and/or bronchiolitis.

The primary outcome was the percentage of PICU admissions born preterm, and secondary outcomes were observed relative risks of invasive mechanical ventilation and mortality within PICU.

We used the Joanna Briggs Institute Checklist for Analytical Cross-Sectional Studies to assess risk of bias.

**Results:**

We included 31 studies, from 16 countries, including a total of 18,331 children.

Following meta-analysis, the pooled estimate for percentage of PICU admissions for RSV/bronchiolitis who were born preterm was 31% (95% confidence interval: 27% to 35%). Children born preterm had a greater risk of requiring invasive ventilation compared to children born at term (relative risk 1.57, 95% confidence interval 1.25 to 1.97, I^2^ = 38%). However, we did not observe a significant increase in the relative risk for mortality within PICU for preterm-born children (relative risk 1.10, 95% confidence interval: 0.70 to 1.72, I^2^ = 0%), although the mortality rate was low across both groups.

The majority of studies (*n* = 26, 84%) were at high risk of bias.

**Conclusions:**

Among PICU admissions for bronchiolitis, preterm-born children are over-represented compared with the preterm birth rate (preterm birth rate 4.4% to 14.4% across countries included in review). Preterm-born children are at higher risk of mechanical ventilation compared to those born at term.

**Supplementary Information:**

The online version contains supplementary material available at 10.1186/s12887-023-04150-7.

## Background

Bronchiolitis is a viral lower respiratory tract infection, which affects babies and young children, most commonly caused by respiratory syncytial virus (RSV) [1]. Bronchiolitis is a clinical diagnosis, although laboratory testing for RSV may be informative. Severe infection in young children may cause apnoea and respiratory failure, and remains an important reason for admission to paediatric intensive care units (PICUs) in those children aged under two years [1]. Providing treatment for respiratory tract infections forms a large part of PICU activity: 30% of PICU admissions in the United Kingdom are due to respiratory causes [2].

Children born preterm are at increased risk of chronic lung disease which in turn can have longer-lasting effects, including increased risk of hospitalisation and severe complications from bronchiolitis [1, 3]. Whilst previous individual studies have examined the proportion of PICU admissions with bronchiolitis in children born preterm and suggested they may be over-represented in PICU, meta-analysis has not been undertaken to date. This issue is gaining importance given the increasing survival following extreme preterm birth which may impact the population of children born preterm requiring PICU services [4], as these children have the highest risk of chronic lung disease [5].

Moreover, individual studies have suggested that children born preterm with bronchiolitis are at higher risk of adverse outcomes within PICU such as requiring invasive ventilation or mortality compared to those born at term [6–10]. The results of these multiple small studies have not yet been pooled to confirm these associations.

The aim of this review was to quantify the burden of preterm birth on PICU admissions for bronchiolitis. We performed a systematic review with meta-analysis, aiming to draw together the evidence for the first time, describing the proportion of PICU admissions for bronchiolitis born preterm, and examining their outcomes within PICU.

## Methods

### Data sources

We searched Medline, Embase and Scopus for articles published from the year 2000 onwards, using search terms for preterm birth, low birth weight or chronic lung disease (proxies for prematurity), and paediatric intensive care [See Additional File for search strategy]. We searched citations and references from the studies included, and screened references from relevant systematic reviews found during the search. This study forms part of a wider over-arching project examining prematurity and PICU. All results from the search were screened and categorised by PICU admission diagnosis, and we present data from studies examining PICU admission for bronchiolitis and/or RSV.

### Study selection

We included cross-sectional and longitudinal studies examining children 0–18 years of age admitted to PICU with RSV and/or bronchiolitis from the year 2000 onwards, taking place in high-income countries as defined by the World Bank [11]. We included only high-income countries to reduce heterogeneity of results, and because these countries are more likely to have a well-established PICU service able to offer intensive care to all children requiring intensive support for respiratory failure. In addition, high-income countries consistently offer neonatal intensive care to the most preterm babies providing more comparable populations of preterm born infants for comparison in this review.

We included studies describing our primary outcome of the proportion of children within PICU born preterm (as per the individual study’s authors’ definition of prematurity), where it was possible to extract these data. We intended to examine secondary outcomes if sufficient data was available: invasive mechanical ventilation, mortality within PICU, repeat admission to PICU, and length of stay in PICU.

Preterm birth is defined by the World Health Organisation as birth before 37 weeks of gestation, and therefore we assumed study authors used this definition unless otherwise specified. Where possible, we examined predefined subgroups by degree of prematurity (< 28 weeks, 28 to < 32 weeks, 32 to < 37 weeks).

To focus on the overall PICU population, we excluded studies which examined *only* children receiving specific therapies within PICU (e.g., extra-corporeal membrane oxygenation), studies examining paediatric intensive care transport, and studies examining high dependency units. We excluded studies which examined *only* children with specific chronic conditions (e.g., congenital heart disease) as these may have different risk factors for PICU admission and adverse outcomes compared to the general PICU population.

To avoid potentially double counting children in studies which used overlapping populations, we included the study reporting the largest sample size. If two studies had overlap but a secondary outcome was only reported in the smaller study, then both the primary outcome from the larger study and the secondary outcome from the smaller study were included.

Initial screening was performed by TvH, with 10% of the primary search screening independently verified by the second reviewer (KW). A third party (SES) was available if disagreements were not resolved after discussion.

### Data extraction

We used Endnote 20 (Clarivate, London UK, 2021), Rayyan (Rayyan Systems Inc, Cambridge USA, 2022), and RevMan 5.41 (Cochrane Collaboration, London UK, 2020) for data management.

We collected the following data items: study characteristics, number of children in PICU born preterm and at term, and number of events for secondary outcomes in preterm-born and term-born groups.

The review was registered on PROSPERO (CRD42021289692) and amendments submitted.

### Data synthesis

We performed data synthesis and meta-analysis in Stata 17 (StataCorp. College Station TX USA, 2021) for outcomes if there were five or more comparable studies, using random-effects models (DerSimonian and Laird), and presented as forest plots. For the primary outcome, we summarised the percentage and 95% confidence intervals (95%CI) of children in PICU born preterm. For secondary outcomes, we used observed data to calculate unadjusted relative risks and 95% confidence intervals for preterm-born compared to term-born children. We reported I^2^ as the measure of heterogeneity.

Studies were included if they reported relevant data to answer our study aim, and we performed critical appraisal based on our research question (irrespective of the aims of the individual study’s authors). For critical appraisal we used the Joanna Briggs Institute Checklist for Analytical Cross-Sectional Studies [12], domains included: inclusion criteria, description of subjects and setting, measurement of exposure (identifying history of preterm birth), confounding factors, measurement of outcomes, and statistical approach. We assigned studies an overall risk of bias: high risk of bias if there were at least two domains with high risk of bias; uncertain if at least two domains had uncertain risk of bias; otherwise, low overall risk of bias. We did not exclude studies on the basis of quality but used it to inform our discussion and interpretation of results.

## Results

**Fig. 1:**
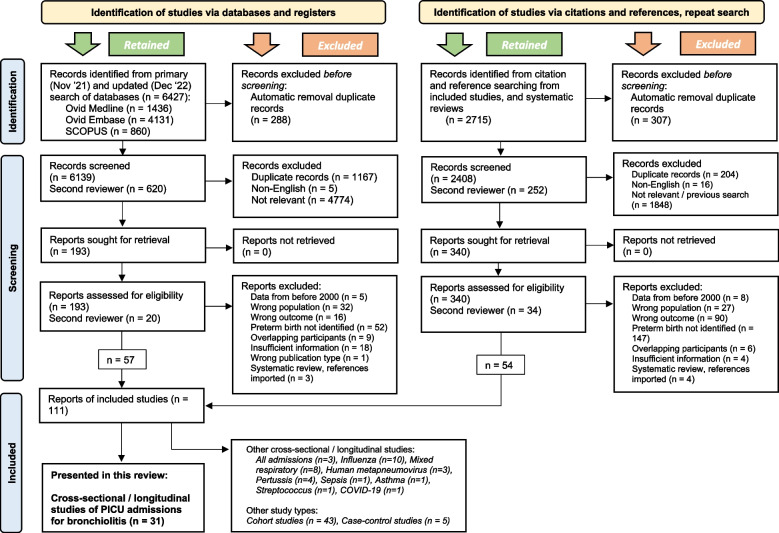
PRISMA flow diagram

We conducted our primary search in November 2021, identifying 5,355 articles, then repeated the search in December 2022, which identified an additional 1,072 abstracts (total *n* = 6,427) (Fig. 1). We excluded non-English language studies (*n* = 5), duplicates (*n* = 1,455), and non-relevant abstracts (*n* = 4,774). Reasons abstracts were considered not relevant were as follows: wrong study design (*n* = 1,534) such as case studies; wrong population (*n* = 1,268), such as low-income countries; wrong outcome (*n* = 877) such as hospitalisation; wrong publication type (*n* = 823) such as commentaries; not examining prematurity (*n* = 152); data from before 2000 (*n* = 84); animal or in-vitro study (*n* = 36).

Following exclusions, we reviewed 193 full studies. Following full review, we excluded further studies for reasons including an inability to identify if the children were born preterm (*n* = 52), or wrong outcome (*n* = 16) such as using a composite outcome of PICU or high dependency care admission.

We screened an additional 2,715 abstracts obtained from citations and references of included studies, resulting in a further 340 papers to review. The same process of inclusion and exclusion of abstracts and full papers was applied.

This search was undertaken as part of a wider study of children admitted to PICU for multiple reasons; for this paper we searched within the wider search results and selected all relevant studies focussing on RSV/bronchiolitis. In total we included 31 studies of PICU admissions for RSV/bronchiolitis in this paper.

### Study characteristics

The 31 studies [6–10, 13–38] were from 16 different countries and included a total of 18,331 children. Data from 28 studies were used for the primary outcome, and from 9 and 7 studies for the secondary outcomes of mortality and ventilation respectively. Characteristics of included studies are summarised in Table 1. There was considerable heterogeneity in study settings, ranging from single centres [9] to national datasets, [28, 29, 31, 34] and units ranged in size from a 7-bed PICU [9] to a 32-bed mixed cardiac/medical PICU [30]. Generally sample sizes were small: 17 studies (57%) included under 200 children, only 2 included over 1,000 [29, 34].

**Table 1:** Characteristics of 31 included studies

**Author**	**Period of inclusion**	**Country**	**National estimate births < 37 weeks**	**Sample Size**	**Preterm, n (%)**	**Setting**	**Age criteria (years)**	**Reason for admission**	**Exclusions: medical conditions**	**Other relevant exclusions**	**Study definition of prematurity**
*Aljassim 2022* [13]	2016–2018	Canada	8.2% (2014)	372	65 (17.5%)	3 hospitals	≤ 2	Suspected/confirmed bronchiolitis	Included	None	Undefined
*Al-Muhsen 2010* [9]	2003–2009	Saudi Arabia	4.4% (2005)	70	26 (37.1%)	1 hospital	Unknown	RSV + bronchiolitis	Included	None	< 36 weeks
*Butt 2011* [14]	2003–2009	Canada	7.6% (2005)	181	73 (40.3%)	1 hospital	< 18	RSV LRTI	Included	None	< 37 weeks
*Carroll 2016* [15]^a^	2009–2011	USA	11.9% (2010)	323	89 (27.6%)	4 hospitals	< 2	RSV + bronchiolitis	Included	None	Undefined
*Essouri 2017* [16]	2013–2014	France and Canada	8.4% (2014) and 8.2% (2014)	194	48 (24.7%)	2 hospitals	< 2	Bronchiolitis	Included	None	Undefined
*Flores-González 2017* [17]	2014–2015	Spain	6.5% (2014)	262	55 (21.0%)	16 hospitals	< 2	Bronchiolitis	Included	Previous episodes of respiratory distress	Undefined
*Ghazaly 2018* [18]	2011–2016	UK	7.1% (2014)	274	126 (46.0%)	1 hospital	< 2	Bronchiolitis	Included	None	< 38 weeks
*Ghazaly 2021* [19]	2016–2018	UK	7.1% (2014)	144	62 (43.1%)	1 hospital	< 2	Bronchiolitis	Excludes respiratory disease and intra-cardiac shunts	None	Undefined
*Guillot 2018* [20]	2013–2015	France	8.4% (2014)	102	35 (34.3%)	1 hospital	< 2	Bronchiolitis	Included	None	Undefined
*Hasagawa 2015* [21]	2007–2010	USA	11.9% (2010)	225	63 (28.0%)	16 hospitals	< 2	Bronchiolitis	Included	Hypernatraemia > 145, no sodium measurement	< 37 weeks
*Huguet 2021* [22]	2010–2018	France	8.4% (2014)	805	223 (27.7%)	1 hospital	< 0.25	Bronchiolitis	Excluded neonatal seizures, hypoxic ischaemic encephalopathy, congenital brain abnormalities, congenital heart disease	None	< 37 weeks
*Javouhey 2008* [23]	2003–2005	France	7.6% (2005)	80	39 (48.8%)	1 hospital	< 1	Bronchiolitis	Excluded tracheostomy, laryngotracheomalacia	NIV used only after extubation	Undefined
*Kadmon 2020* [24]	2012–2016	Israel	8.6% (2014)	276	87 (31.5%)	5 hospitals	< 2	RSV + Bronchiolitis	Included	None	< 37 weeks
*Kang 2019* [7]	2008–2013	South Korea	5.7% (2010)	92	7 (7.6%)	6 hospitals	< 18	RSV	Excluded haematology/oncology conditions, other severe underlying condition causing admission	Elective surgery	< 35 weeks
*Koutsaftiki 2013* [25]	2007–2012	Greece	10.7% (2010)	120	36 (30.0%)	2 hospitals	Unknown	Bronchiolitis	Included	None	< 36 weeks
*Lee 2016* [26]	2001–2010	Taiwan	-	186	92 (49.5%)	1 hospital	< 18	RSV LRTI	Included	None	< 37 weeks
*Lee 2021* [27]	2004–2010	Singapore	10.4% (2014)	85	40 (47.1%)	1 hospital	Unknown	RSV	Included	None	Undefined
*Leung 2014* [28]	2009–2011	Hong Kong	-	118	35 (29.7%)	8 hospitals—all PICUs in country	< 18	RSV	Included	None	< 37 weeks
*Linssen 2021* [29]	2003–2016	Netherlands	7.5% (2010)	2161	564 (26.1%)	8 hospitals—all PICUs in country	< 3	RSV + Bronchiolitis	Included	None	< 37 weeks
*Marlow 2021* [30]	2015–2019	USA	9.6% (2014)	573	195 (34.0%)	1 hospital	< 2	Bronchiolitis	Excludes single ventricle physiology and long term ventilation	Never requiring invasive or non-invasive ventilation	< 37 weeks
*McKiernan 2010* [10]	2005–2007	USA	11.7% (2005)	115	34 (29.6%)	1 hospital	< 2	Bronchiolitis	Excludes tracheostomy	Pre-hospital intubation	< 37 weeks
*Pham 2020* [6]^b^	2005–2015	Australia	7.9% (2010)	604	103 (17.1%)	1 hospital	Unknown	RSV	Included	Hospital acquired RSV, post-operative admissions	Undefined
*Prais 2003* [8]^c^	2000–2001	Israel	14.4% (2000)	104	32 (30.8%)	11 hospitals	Unknown	RSV + bronchiolitis	Included	None	< 37 weeks
*Prais 2005* [31]	2000–2002	Israel	14.4% (2000)	228	94 (41.2%)	13 hospitals—national PICU network	Unknown	RSV + Bronchiolitis	Included	None	< 37 weeks
*Resch 2018* [32]	2006–2015	Austria	8.5% (2010)	156	52 (33.3%)	1 hospital	Unknown	RSV	Included	None	Undefined
*Schiller 2011* [33]	2004–2007	Israel	11.9% (2005)	79	31 (39.2%)	1 hospital	< 2	RSV + Bronchiolitis	Excludes immunodeficiency, transplant, malignancy	None	< 37 weeks
*Schlapbach 2017* [34]	2002–2014	Australia and New Zealand	7.9% (2010) and 8.0% (2010)	9304	1697 (18.2%)	19 hospitals—national registries	< 2	Bronchiolitis	Excludes tracheostomies	Elective admissions	< 37 weeks
*Slain 2018* [35]	2013–2014	USA	9.6% (2014)	145	45 (31.0%)	1 hospital	< 2	Bronchiolitis	Included	None	< 37 weeks
*Soilly 2012* [36]	2005–2006	France	7.6% (2005)	467	149 (31.9%)	24 hospitals	< 2	Bronchiolitis	Included	None	< 37 weeks
*Soshnick 2019* [37]	2010–2012 and 2015–2016	USA	9.6% (2014)	325	57 (17.5%)	1 hospital	< 2	Bronchiolitis	Included	None	< 34 weeks
*Toni 2019* [38]	2010–2011 and 2016–2017	Spain	6.5% (2014)	161	29 (18.0%)	1 hospital	< 1	Bronchiolitis	Excludes tracheostomies, previous wheeze or bronchiolitis	None	Undefined

National preterm birth estimates from: Chawanpaiboon S, Vogel JP, Moller A-B, et al. Global, regional, and national estimates of levels of preterm birth in 2014: a systematic review and modelling analysis. Lancet Glob Health. 2019;7:e37-e46 Online. [Interactive tables available from: https://ptb.srhr.org/. Accessed 27.10.2022.]

^a^Overlapping study population with Soshnick 2019 [37]

^b^Overlapping study population with Schlapbach 2017 [34]

^c^Overlapping study population with Prais 2005 [31]

The majority of studies (17/31, 55%) included children with a clinical diagnosis of bronchiolitis, there were seven which included children with a positive test for RSV, and seven in which the inclusion criteria was clinicial bronchiolitis with confirmed RSV (23% each) (Table 1). The majority of studies included children up to the age of two years; one included only infants up to three months of age [22], and four included children up to 18 years [7, 14, 26, 28], although the majority of participants were aged under one year regardless of inclusion criteria. Seven (23%) studies did not describe inclusion criteria for age.

Eleven studies did not describe the threshold for preterm birth used and therefore this was assumed to be < 37 weeks. Fifteen studies specified preterm birth as < 37 weeks, one study used < 38 weeks [18], the remaining four used between < 34 and < 36 weeks [7, 9, 25, 37]. Where lower thresholds were used, studies may have included a smaller number of more preterm children who are at greater risk of chronic health conditions.

### Study quality

**Fig. 2:**
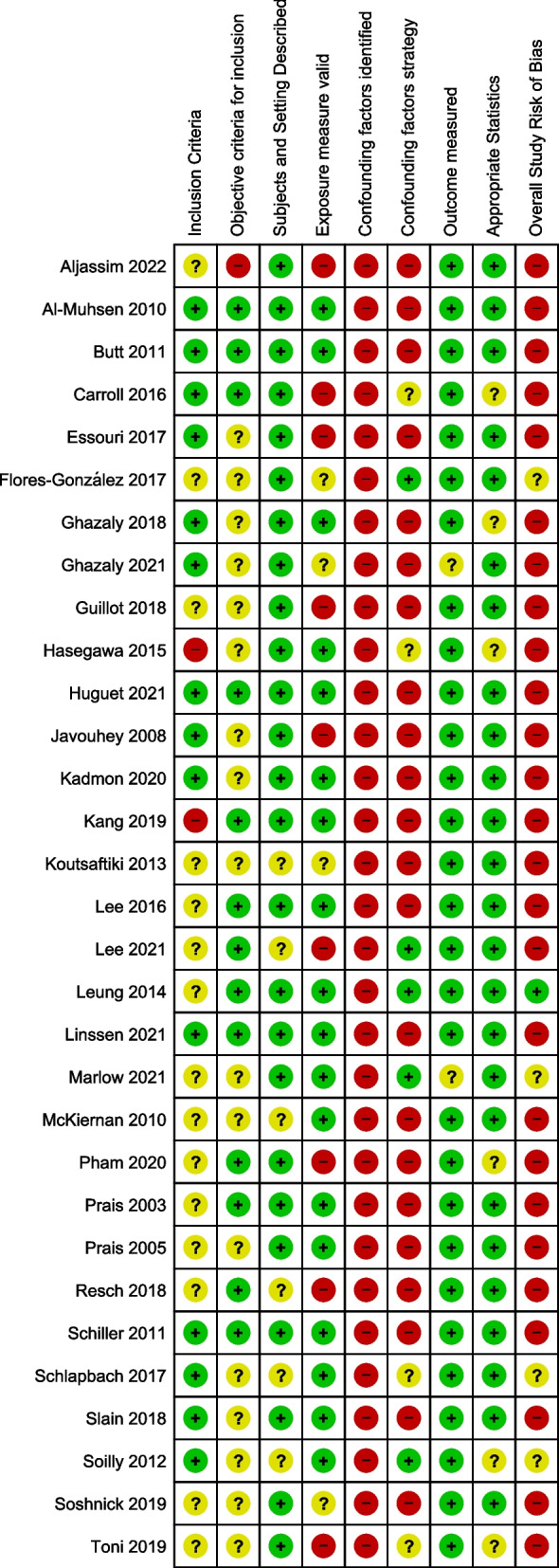
Risk of bias summary for included studies

Figure 2 shows a summary of the risk of bias assessments for the 31 studies, of which 26 (84%) had a high risk of bias, four (13%) had an unclear risk of bias [17, 30, 34, 36], and one (3%) had a low risk of bias [28]. No studies compared confounding factors such as comorbidities between preterm and term-born groups, so all were assigned a high risk of bias in this domain. Other issues identified included the definition of preterm birth; and exclusion of children for reasons not relevant to this review such as hypernatremia [21] or pre-hospital intubation [10].

### Primary outcome

**Fig. 3:**
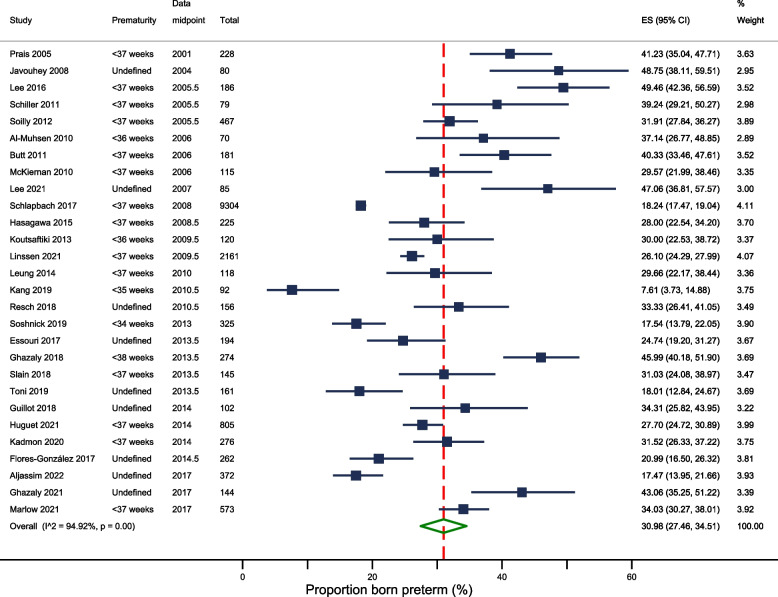
Forest plot: proportion of children born preterm among PICU admissions for RSV and/or bronchiolitis

Meta-analysis of 28 studies (after excluding three studies with overlapping populations [6, 8, 15]) gave a pooled estimate for the percentage of children admitted to PICU with RSV and/or bronchiolitis born preterm of 31.0% (95%CI: 27.5% to 34.5%) (Fig. 3). However, there was significant heterogeneity (I^2^ = 95%). After excluding studies in which prematurity was not defined [16, 17, 19, 20, 23, 27, 32, 38], the pooled estimate remained similar at 31.0% (95%CI: 26.7% to 35.3%), but heterogeneity remained high (I^2^ = 96%).

There appeared to be changes over time with a higher percentage of PICU admissions born preterm in earlier studies. For studies whose midpoint of data collection was before 2010 – the year the Joint Committee on Vaccination and Immunisation (JCVI) recommended use of the RSV prophylaxis in the United Kingdom (UK) [39] – the percentage of children born preterm was 35.3% (95%CI: 29.7% to 40.9%); for studies whose midpoint was ≥ 2010, the percentage born preterm was 27.5% (95%CI: 22.6% to 32.5%).

The pooled percentage of PICU admissions born extremely preterm (reported in five studies [7, 18, 30, 31, 33]) was 5.0% (range: 1.3% to 11.0%, 95%CI: 2.0% to 8.1%) [see Additional File for forest plot], with significant heterogeneity between studies (I^2^ = 83%). There was no clear relationship between the study period and the proportion of children born extremely preterm.

### Secondary outcomes

Meta-analysis for secondary outcomes was performed for mortality in PICU (nine studies) and invasive ventilation (seven studies). However, there were no studies reporting repeat PICU admission, and only three reported length of stay [7, 17, 28] so these outcomes are not presented.

**Fig. 4:**
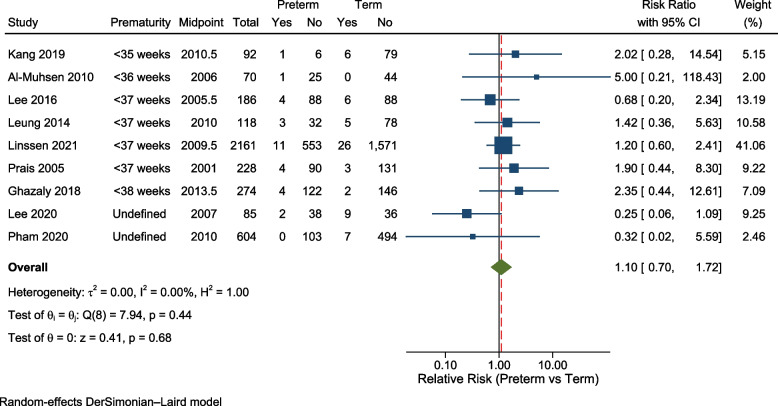
Forest plot: relative risk of mortality within PICU comparing children born preterm and at term

The overall crude relative risk for mortality within PICU, using data from nine studies, was 1.10 (95%CI: 0.70 to 1.72), with a lack of heterogeneity (I^2^ = 0%) (Fig. 4). To examine publication bias, we examined the funnel plot for this outcome (not shown), which appeared symmetrical (Egger’s test *p* = 0.81). Sensitivity analysis was performed, excluding the two studies that did not define prematurity [6, 27], and meta-analysis of the remaining seven studies gave a relative risk for mortality of 1.33 (95%CI: 0.82 to 2.13) (I^2^ = 0%).

To examine whether the introduction of RSV prophylaxis may have affected mortality, we performed subgroup analysis of studies whose midpoints were before 2010 and those who midpoint was 2010 or after. For the five earlier studies the relative risk of observed mortality was 0.94 (95%CI: 0.47 to 1.89), and for the four studies with a data midpoint from 2010 the relative risk was 1.52 (95%CI 0.63 to 3.71).

**Fig. 5:**
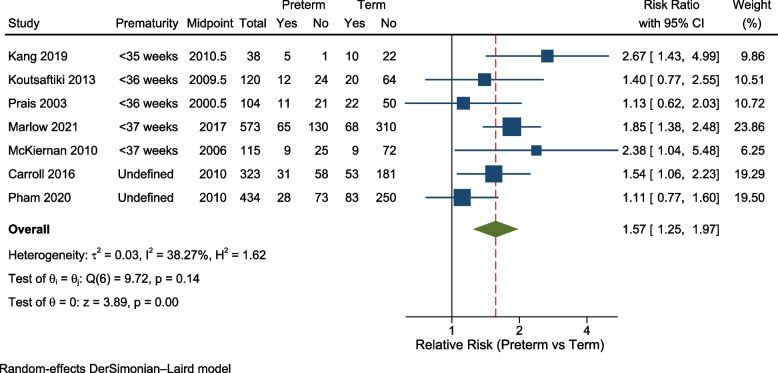
Forest plot: relative risk of invasive ventilation comparing children born preterm and at term

Meta-analysis using seven studies showed children born preterm had 1.57 times (95%CI: 1.25 to 1.97) the crude relative risk of ventilation compared to children born at term (Fig. 5). There was low to moderate heterogeneity (I^2^ = 38%). The funnel plot (not shown) appeared symmetrical (Egger’s test *p* = 0.51). We again performed sensitivity analysis excluding two studies that did not define prematurity [6, 15], which gave a relative risk for ventilation of 1.76 (95%CI: 1.35 to 2.30) (I^2^ = 21%), confirming a consistent increase in risk.

In relation to RSV prophylaxis, examining the three studies whose data midpoint was before 2010, the relative risk for ventilation was 1.43 (95%CI: 0.98 to 2.10), and for the four later studies it was 1.63 (95%CI: 1.20 to 2.21).

## Discussion

In this review we aimed to quantify the burden of prematurity within the PICU whilst focussing on a specific clinical condition associated with severe complications in children born preterm: bronchiolitis. We identified that the pooled percentage of children admitted to PICU for RSV/bronchiolitis born preterm (31%) is consistently higher than would be expected from the rate of preterm births in countries included in this review (range: 4.4% in Saudi Arabia to 14.4% in Israel [40], Table 1). Where reported, the pooled percentage of children born extremely preterm (5.0% of admissions) was also greater than the extreme preterm live birth rate (for example 0.5% of live births in the UK [41]).

The observed mortality following PICU admission for bronchiolitis was very low in both preterm- and term-born children, with no significant difference shown. Given the low observed frequencies of deaths from bronchiolitis, it may be that a larger study population is required to avoid type II error. However, our results demonstrate that preterm-born children are at 1.5 times the risk of requiring ventilation compared to those born at term, demonstrating the need for increased intensity of care. Therefore, it appears preterm-born children have higher needs of medical intervention to successfully recover from bronchiolitis.

Over the years of our study, the practice of passive immunisation against RSV for high-risk preterm-born infants has become standard across high-income countries. RSV prophylaxis is known to reduce the risk of hospitalisation for bronchiolitis [42], although the effect on overall PICU demand remains unknown. To explore this further as a sensitivity analysis, we examined admissions before and after 2010, using the publication of UK JCVI recommendations for RSV prophylaxis as a threshold [39]. Despite decreased neonatal mortality following extreme preterm birth during this period [4] (and therefore more surviving children), later studies reported a lower percentage of children admitted to PICU who were born preterm, although confidence intervals did overlap. Therefore, RSV prophylaxis may have reduced PICU admissions for bronchiolitis in preterm-born children, although we were unable to explore this further as we did not have child-level data describing individuals’ RSV prophylaxis status. Moreover, there was no clear trend in the extremely preterm-born subgroup, who would generally meet criteria for RSV prophylaxis. In addition, changes could be due to other factors over this period such as adoption of high-flow nasal cannula oxygen therapy to avoid PICU admission [6, 10].

We also examined the secondary outcomes comparing studies taking place before and after the introduction of RSV prophylaxis. The relative risk of mortality for preterm-born compared to term-born children appeared to be increased in the later studies, however the confidence intervals were very wide as there were only a limited number of studies, and death in PICU was infrequent, and so this result may be due to chance. For ventilation, the relative risk was greater in the later studies, this could reflect differential effects of advances in respiratory management between the term-born and preterm-born children, resulting in a relatively greater proportion of those born preterm requiring ventilation while more term-born children could be managed using non-invasive therapies. However, there were again few studies in each sub-group, and so it is difficult to draw firm conclusions.

### Strengths and weaknesses

This novel systematic review answers an important research question for a vulnerable group of children. Its strengths lie in a wide-ranging analysis and summary of many smaller studies, and using a broad systematic search strategy – including screening of references and citations – to reduce the chance of missing relevant studies. Moreover, second review of a proportion of studies took place throughout, including at data extraction and critical appraisal stages to ensure consistency in the inclusion approach. Appropriate random-effects models were used due to heterogeneity across studies. This heterogeneity may have arisen from a range of factors such as variation in inclusion criteria for age, whether RSV test positivity was required for inclusion, and variation in PICU size across included studies. There were studies that included all children under 18 years of age, a range which does not reflect the age distribution of RSV/bronchiolitis where the majority of severely affected children are aged under 2 years [1].

Limitations include a lack of evidence directly addressing the specified research question, and consequently due to paucity of data, true differences may have been missed. Many studies did not have prematurity as the primary focus, and therefore the majority of studies were assigned a high risk of bias. Of note, studies did not compare the presence of other risk factors for adverse PICU outcomes such as comorbidities between preterm- and term-born groups. Therefore, further sensitivity analysis or meta-regression was limited by the lack of data, and we could only calculate observed crude unadjusted relative risks.

### Future work

There is a lack of evidence addressing this important research question and limited data describing the full impact of preterm birth on PICU services, or outcomes for preterm-born children in PICU. Understanding the impact of preterm birth is important for families, clinicians, and policy makers. Contemporary large high-quality studies are needed to accurately plan PICU services and inform parents of preterm children of the risks of further critical illness following neonatal care. We intend to use the UK national-level dataset of PICU admissions (PICANet [2]) to further answer these questions in the future.

## Conclusions

Admission to paediatric intensive care of preterm-born children impacts children, their families, and the wider healthcare system. Preterm-born children make up a significant and over-represented group within the children admitted to PICU with bronchiolitis. They are at higher risk of requiring invasive respiratory support than children born at term, but no significant increased risk of mortality was observed.

## Supplementary Information

**Additional file 1. Supplementary Table.** Search strategy. **Supplementary Figure**. Forest plot showing the proportion of children born at <28 or ≤28 weeks among paediatric intensive care admissions for respiratory syncytial virus and/or bronchiolitis.

## Data Availability

The corresponding author may be contacted to share the data and materials of this review subject to reasonable requests.

## Abbreviations

95% CI: 95% Confidence interval
ES: Effect size
JCVI: Joint Committee on Vaccination and Immunisation, United Kingdom
LRTI: Lower respiratory tract infection
NIV: Non-invasive ventilation
PICU: Paediatric intensive care unit
RSV: Respiratory syncytial virus
UK: United Kingdom
USA: United States of America

## References

[1] Øymar K, Skjerven HO, Mikalsen IB (2014). Acute bronchiolitis in infants, a review. Scand J Trauma Resusc Emerg Med.

[2] PICANet. PICANet Annual Reporting and Publications: PICANet; 2021. Available from: https://www.picanet.org.uk/annual-reporting-and-publications/. Accessed 18 Oct 2021.

[3] McLaurin KK, Farr AM, Wade SW (2016). Respiratory syncytial virus hospitalization outcomes and costs of full-term and preterm infants. J Perinatol.

[4] British Association of Perinatal Medicine. Perinatal Management of Extreme Preterm Birth Before 27 weeks of Gestation (2019). A BAPM Framework for Practice 2019 [updated 23/10/2019]. Available from: https://www.bapm.org/resources/80-perinatal-management-of-extreme-preterm-birth-before-27-weeks-of-gestation-2019. Accessed 12 Oct 2021.

[5] KalikkotThekkeveedu R, Guaman MC, Shivanna B (2017). Bronchopulmonary dysplasia: a review of pathogenesis and pathophysiology. Respir Med.

[6] Pham H, Thompson J, Wurzel D (2020). Ten years of severe respiratory syncytial virus infections in a tertiary paediatric intensive care unit. J Paediatr Child Health.

[7] Kang JM, Lee J, Kim YK (2019). Pediatric intensive care unit admission due to respiratory syncytial virus: retrospective multicenter study. Pediatr Int.

[8] Prais D, Schonfeld T, Amir J (2003). Admission to the intensive care unit for respiratory syncytial virus bronchiolitis: a national survey before palivizumab use. Pediatrics.

[9] Al-Muhsen SZ (2010). Clinical profile of Respiratory Syncytial Virus (RSV) bronchiolitis in the intensive care unit at a tertiary care hospital. Curr Pediatr Res.

[10] McKiernan C, Chua LC, Visintainer PF (2010). High flow nasal cannulae therapy in infants with bronchiolitis. J Pediatr.

[11] The World Bank. World Bank Country and Lending Groups 2022. Available from: https://datahelpdesk.worldbank.org/knowledgebase/articles/906519-world-bank-country-and-lending-groups. Accessed 25 Feb 2022.

[12] JBI. Critical Appraisal Tools: Faculty of Health and Medical Sciences at the University of Adelaide, South Australia; 2022. Available from: https://jbi.global/critical-appraisal-tools. Accessed 25 Feb 2022.

[13] Aljassim NA, Noël KC, Maratta C (2022). Antimicrobial Stewardship in Bronchiolitis: A Retrospective Cohort Study of Three PICUs in Canada. Pediatr Crit Care Med.

[14] Butt ML, Symington A, Janes M (2011). The impact of prophylaxis on paediatric intensive care unit admissions for RSV infection: a retrospective, single-centre study. Eur J Pediatr.

[15] Carroll CL, Faustino EVS, Pinto MG (2016). A regional cohort study of the treatment of critically ill children with bronchiolitis. J Asthma.

[16] Essouri S, Baudin F, Chevret L (2017). Variability of care in infants with severe bronchiolitis: less-invasive respiratory management leads to similar outcomes. J Pediatr.

[17] Flores-González JC, Mayordomo-Colunga J, Jordan I (2017). Prospective Multicentre Study on the Epidemiology and Current Therapeutic Management of Severe Bronchiolitis in Spain. Biomed Res Int.

[18] Ghazaly M, Nadel S (2018). Characteristics of children admitted to intensive care with acute bronchiolitis. Eur J Pediatr.

[19] Ghazaly MM, Abu Faddan NH, Raafat DM (2021). Acute viral bronchiolitis as a cause of pediatric acute respiratory distress syndrome. Eur J Pediatr.

[20] Guillot C, Le Reun C, Behal H (2018). First-line treatment using high-flow nasal cannula for children with severe bronchiolitis: Applicability and risk factors for failure. Arch Pediatr.

[21] Hasegawa K, Stevenson MD, Mansbach JM (2015). Association between hyponatremia and higher bronchiolitis severity among children in the ICU with bronchiolitis. Hosp Pediatr.

[22] Huguet A, Valla F, Toulouse J (2021). Occurrence and risk factors associated with seizures in infants with severe bronchiolitis. Eur J Pediatr.

[23] Javouhey E, Barats A, Richard N (2008). Non-invasive ventilation as primary ventilatory support for infants with severe bronchiolitis. Intensive Care Med.

[24] Kadmon G, Feinstein Y, Lazar I (2020). Variability of care of infants with severe respiratory syncytial virus bronchiolitis: a multicenter study. Pediatr Infect Dis J.

[25] Koutsaftiki C, Tsialla A, Sideri G, Bampanelou A, Papadatos I, Sianidou L (2013). Clinical data of infants with bronchiolitis who are admitted to two picus. Intensive Care Med.

[26] Lee Y-I, Peng C-C, Chiu N-C (2016). Risk factors associated with death in patients with severe respiratory syncytial virus infection. J Microbiol Immunol Infect.

[27] Lee MW, Goh AE (2021). Mortality in children hospitalised with respiratory syncytial virus infection in Singapore. Singapore Med J.

[28] Leung T, Lam D, Miu T (2014). Epidemiology and risk factors for severe respiratory syncytial virus infections requiring pediatric intensive care admission in Hong Kong children. Infection.

[29] Linssen RS, Bem RA, Kapitein B (2021). Burden of respiratory syncytial virus bronchiolitis on the Dutch pediatric intensive care units. Eur J Pediatr.

[30] Marlow RK, Brouillette S, Williams V (2021). Risk Factors Associated with Mechanical Ventilation in Critical Bronchiolitis. Children.

[31] Prais D, Danino D, Schonfeld T (2005). Impact of palivizumab on admission to the ICU for respiratory syncytial virus bronchiolitis: a national survey. Chest.

[32] Resch B, Brunner K, Rödl S (2018). Characteristics of severe RSV infection needing intensive care. Eur J Pediatr.

[33] Schiller O, Levy I, Pollak U (2011). Central apnoeas in infants with bronchiolitis admitted to the paediatric intensive care unit. Acta Paediatr.

[34] Schlapbach LJ, Straney L, Gelbart B (2017). Burden of disease and change in practice in critically ill infants with bronchiolitis. Eur Respir J.

[35] Slain KN, Shein SL, Stormorken AG (2018). Outcomes of children with critical bronchiolitis living in poor communities. Clin Pediatr (Phila).

[36] Soilly A-L, Ferdynus C, Desplanches O (2012). Paediatric intensive care admissions for respiratory syncytial virus bronchiolitis in France: results of a retrospective survey and evaluation of the validity of a medical information system programme. Epidemiol Infect.

[37] Soshnick SH, Carroll CL, Cowl AS (2019). Increased use of noninvasive ventilation associated with decreased use of invasive devices in children with bronchiolitis. Crit Care Explor.

[38] Toni F, Lasaosa FJC, Conti G (2019). Comparison in the management of respiratory failure due to bronchiolitis in a pediatric ICU between 2010 and 2016. Respir Care.

[39] Joint Committee on Vaccination and Immunisation. Joint Committee on Vaccination and Immunisation Statement on immunisation for Respiratory Syncytial Virus 2010 [updated 7/01/2013]. Available from: https://webarchive.nationalarchives.gov.uk/ukgwa/20130107105354/http://www.dh.gov.uk/prod_consum_dh/groups/dh_digitalassets/@dh/@ab/documents/digitalasset/dh_120395.pdf. Accessed 26 Oct 2022.

[40] Chawanpaiboon S, Vogel JP, Moller A-B (2019). Global, regional, and national estimates of levels of preterm birth in 2014: a systematic review and modelling analysis. Lancet Glob Health.

[41] Office for National Statistics. Dataset: Birth Characteristics 2020 edition 2022 [updated 13/01/2022]. Available from: https://www.ons.gov.uk/peoplepopulationandcommunity/birthsdeathsandmarriages/livebirths/datasets/birthcharacteristicsinenglandandwales. Accessed 01 Mar 2023.

[42] The IMpact-RSV Study Group (1998). Palivizumab, a Humanized Respiratory Syncytial Virus Monoclonal Antibody, Reduces Hospitalization From Respiratory Syncytial Virus Infection in High-risk Infants. Pediatrics.

