# Supplementary material for: Children born preterm admitted to paediatric intensive care for bronchiolitis: a systematic review and meta-analysis
Source: BMC Pediatr. 2023 Jun 29;23:326. doi: 10.1186/s12887-023-04150-7 (PMC10308614; doi:10.1186/s12887-023-04150-7)

**Supplementary Table – Search strategy**

| **Ovid MEDLINE** | **Ovid Embase** | **Scopus** |
| --- | --- | --- |
| exp Infant, Premature/ or Infant, Very Low Birth Weight/ or Infant, Extremely Low Birth Weight/ or exp Infant, Premature, Diseases/ or Gestational Age/ or Premature Birth/ | exp prematurity/ or exp very low birth weight/ or exp extremely low birth weight/ or gestational age/ or chronic lung disease/ or lung dysplasia/ | prematur* OR preterm OR "very low birth weight" OR "extremely low birth weight" OR gestation OR "gestational age" OR “chronic lung disease” OR CLD OR "bronchopulmonary dysplasia" OR BPD |
| *OR* | *OR* | *AND* |
| Preterm.mp. or Prematur*.mp. or very low birth weight.mp. or extremely low birth weight.mp. or gestation.mp. or gestational age.mp. or bronchopulmonary dysplasia.mp. or BPD.mp. or Chronic lung disease.mp. or CLD.mp. | prematur*.mp. or preterm.mp. or very low birth weight.mp. or extremely low birth weight.mp. or gestation.mp. or gestational age.mp. or chronic lung disease.mp. or bronchopulmonary dysplasia.mp or CLD.mp. or BPD.mp. | p*ediatric intensive care OR p*ediatric critical care OR PICU |
| *AND* | *AND* | *AND* |
| Critical Care/ or Critical Illness/ or Intensive Care Units, Pediatric/ | intensive care/ or intensive care medicine/ or exp pediatric intensive care unit/ | PUBYEAR > 1999 AND ( LIMIT-TO ( LANGUAGE , "English" ) ) |
| *OR* | *OR* |  |
| p?ediatric intensive care.mp. or PICU.mp. or p?ediatric critical care.mp. | p?ediatric intensive care.mp. or p?ediatric critical care.mp. or PICU.mp. |  |
| *AND* |  |  |
| limit to (English language and humans and yr="2000 -Current") | limit to (human and English language and yr="2000 -Current") |  |

**Supplementary Figure – Forest plot showing the proportion of children born at <28 or ≤28 weeks among paediatric intensive care admissions for respiratory syncytial virus and/or bronchiolitis**


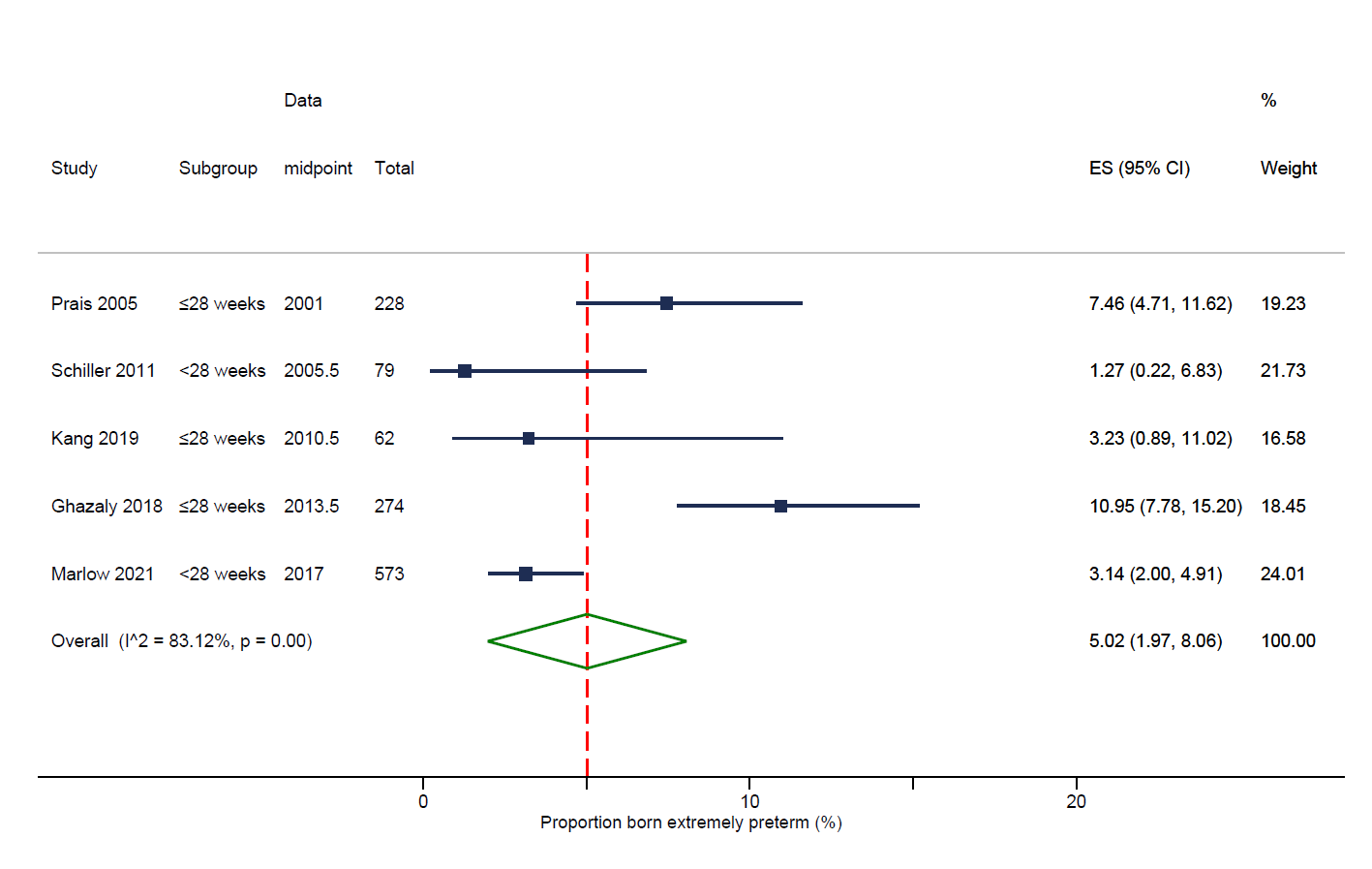

Supplement: Supplementary file 1 — Additional file 1. Supplementary Table. Search strategy. Supplementary Figure. Forest plot showing the proportion of children born at <28 or ≤28 weeks among paediatric intensive care admissions for respiratory syncytial virus and/or bronchiolitis. [file 12887_2023_4150_MOESM1_ESM.docx]
